# 3D Laser Triangulation for Plant Phenotyping in Challenging Environments

**DOI:** 10.3390/s150613533

**Published:** 2015-06-09

**Authors:** Katrine Heinsvig Kjaer, Carl-Otto Ottosen

**Affiliations:** Department of Food Science, Aarhus University, Kirstinebjergvej 10, 5792 Aarslev, Denmark; E-Mail: coo@food.au.dk

**Keywords:** high-throughput phenotyping (HTPP), sensor-to-plant concept, rapeseed (*Brassica napus*), leaf area, shoot biomass, chlorophyll fluorescence, photosystem II activity, growth rate, automated growth measurement, 3D laser scanner

## Abstract

To increase the understanding of how the plant phenotype is formed by genotype and environmental interactions, simple and robust high-throughput plant phenotyping methods should be developed and considered. This would not only broaden the application range of phenotyping in the plant research community, but also increase the ability for researchers to study plants in their natural environments. By studying plants in their natural environment in high temporal resolution, more knowledge on how multiple stresses interact in defining the plant phenotype could lead to a better understanding of the interaction between plant responses and epigenetic regulation. In the present paper, we evaluate a commercial 3D NIR-laser scanner (PlantEye, Phenospex B.V., Herleen, The Netherlands) to track daily changes in plant growth with high precision in challenging environments. Firstly, we demonstrate that the NIR laser beam of the scanner does not affect plant photosynthetic performance. Secondly, we demonstrate that it is possible to estimate phenotypic variation amongst the growth pattern of ten genotypes of *Brassica napus* L. (rapeseed), using a simple linear correlation between scanned parameters and destructive growth measurements. Our results demonstrate the high potential of 3D laser triangulation for simple measurements of phenotypic variation in challenging environments and in a high temporal resolution.

## 1. Introduction

Plant screening and phenotyping technologies with an appropriate resolution in fluctuating climate environments are essential to improve the efficiency of high-throughput plant phenotyping (HTPP) towards understanding how phenotypic variation is linked to environmental conditions. Most sensors enabling non-destructive measurements of plant growth are based on optical principles, which are sensitive to changes in illumination. Optical principles require complicated normalization and calibration software, making their application impracticable, and sometimes impossible in challenging environments. This issue can be easily solved by using closed cabinets to which plants are transported for imaging, or by measuring under dark conditions [1,2]. However, though transport has been shown not to affect plant growth [3], it comes along with lower throughput. Furthermore, in many phenotyping platforms, plants are often placed in rows with large distances between the pots [1,4], or screened individually following a plant-to-sensor concept [2,5]. These applications do not reflect the situation in the field or in production units where plants are grown at high density and where the structure of the individual plant is affected by competition for light and resources. Moreover, diurnal measurements are usually not possible because of insufficient throughput, even though high temporal resolution is a fundamental requirement to understand how external and internal factors determine differences in canopy structure, and how different genotypes adapt to the dynamic and changing environment. For instance, leaves within natural canopies are constantly changing orientation due to endogenous mechanisms and external factors such as light and water availability [6]. Direct methods to quantify these structural changes are important to understand on how plants optimize the canopy structure to maximize light utilization and minimize water loss under heat and drought stress.

The development of 3D imaging techniques for estimating canopy structure, shoot growth and biomass has expanded during the last couple of years. Stereo camera systems using two RGB (red, green, blue) cameras give the ability to capture three-dimensional images [7,8], whereas sensors more suitable for greenhouse and field conditions are light detection and ranging (LIDAR) and laser triangulation [9,10]. In contrast to camera systems which are passive, LIDAR and laser triangulation are active devices, in which a light beam (laser line or dot) is projected onto plants and the energy scattered from the plant is used for the computation of depth maps and 3D point clouds. In a recent paper laser triangulation was used in a lysimetric platform to phenotype transpiration dynamics of thousands of plants [11].

Light sources in the visible spectrum have direct effects on plant photosynthesis and primary carbohydrate metabolism, and affect the circadian clock if applied in dark periods. Furthermore, near-infrared light that is not reflected can induce transitions in the manganese cluster of photosystem II (PSII) above 800 nm [12], and recent findings illustrated that far-red light up to 800 nm can drive PSII electron transport [13], suggesting that photochemistry may also be affected by even longer wavelengths. This can have implications for processes related to the photosynthetic apparatus in plants, and may affect plant yield. However, to our knowledge, no effort has been put into studying these effects when validating the use of light sources in plant growth sensors.

The purpose of the present paper was to evaluate a commercial 3D laser triangulation scanner and to test its suitability for HTPP in a greenhouse environment.

## 2. Material and Methods

### 2.1. The 3D Laser Triangulation Scanner

**Figure 1:**
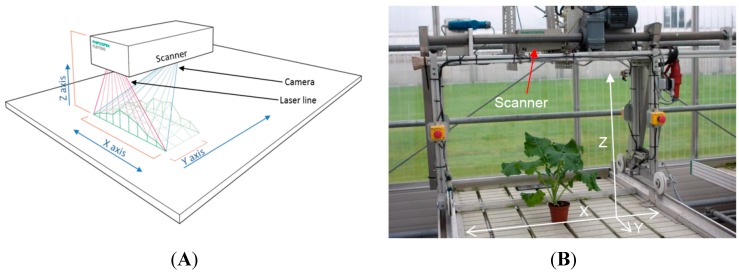
(**A**) Drawing of the 3D scanner, the red lines display the width and projection of the laser line, the blue lines are the projection and width of the camera and the green line/area displays the canopy of a crop stand; (**B**) The boom system in its starting position with the 3D scanner mounted.

The automated plant growth measurements were performed using the commercial 3D laser triangulation scanner PlantEye F300 developed by Phenospex B.V. (Heerlen, the Netherlands) (Figure 1A). The sensor projects a laser line in the near infrared (NIR) region of the light spectrum vertically downwards and captures the scattered light with an integrated CMOS-camera.

A NIR is used to increase data quality since most of the light is reflected from plants. A sunlight filter reduces all artifacts like reflections or background noise from sunlight or other light sources, allowing reproducible measurements under direct sunlight or high irradiances. Moreover, all internal parts like the laser diode and camera is temperature-controlled by a thermoelectric cooler, allowing the operation of the sensor at ambient temperatures of up to 45 °C without cutback or loss of data quality.

The 3D laser triangulation scanner was mounted on a boom (Technical University of Southern Denmark, Odense, Denmark) (Figure 1B). The boom was placed on a greenhouse table (1.2 × 8 m), with the distance of the scanner to the table of 850 mm, resulting in a scan width (x-direction) of 640 mm. The automated boom can be programmed to run at six different velocities (denoted 1–6), and at specific time points during the day and on specific days, controlled by a digital timer system. To check on the evenness of the velocity of the boom, the time was recorded after every 200 mm at velocity 6. The velocity was measured to be 50.4 mm·s^−1^ with a standard error of 1.5 mm·s^−1^ over a distance of 6 m. During the scanning process the scanner moves linearly over the plants along the y-axis of the scanning field on the greenhouse table (Figure 1A, B), and collects images of the projected laser light on the plants.

**Figure 2:**
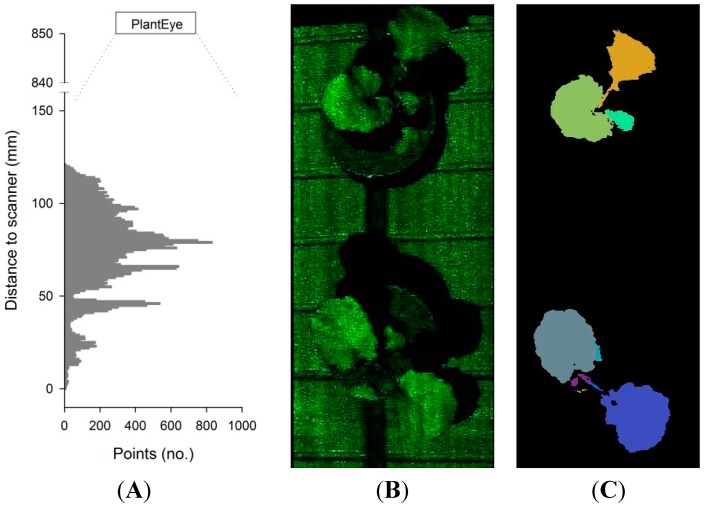
(**A**) Histogram showing the number of points at different distances to the scanner; (**B**) the raw 3D point cloud of two rapeseed plants and (**C**) the segmented 3D point cloud.

The resolution in the y-direction depends on the scanning speed of the scanner, which generates 50 xz depth profiles per second, with a resolution of 0.8 mm in the x-direction (width) and a resolution of 0.2 mm in the z-direction (distance from scanner). For instance, if the scanning speed is 50 mm·s^−1^, the resolution in the y-direction is 1 mm. The scanning field can be divided into a number of subfields, and from each subfield image, depth profiles of the x-z plane are computed. These depth profiles can be arranged as histograms showing the number of points at a specific distance from the scanner (z-direction) (Figure 2A), or displayed as a raw 3D point cloud of the subfield canopy (Figure 2B). From each 3D point cloud, the meshing of neighboring points (segmentation) is automatically carried out (Figure 2C), and plant height, projected leaf area, 3D leaf area and leaf angle distribution of the subfield canopy are computed.

The height of plants placed in each subfield is calculated from the histograms (Figure 2A). The points are arranged in percentiles, in relation to their distance from the scanner, and only a part is used for the computation of the plant height. This process is called cropping and is defined in the sensor settings. In most plant species, 80% of the lower points and 10% of the higher points of the histogram are discarded, as the average of the remaining points (between the 80 to 90% percentiles) has been found to give a robust estimate of plant height. The projected leaf area is calculated based on the segmented leaf area in relation to the subfield area (Figure 2C), whereas the 3D leaf area is computed by considering the distances in 3D taking the steepness of the leaf angles into account. This is a far better estimation of leaf area compared to the projected leaf area which is delivered by 2D imaging.

**Figure 3:**
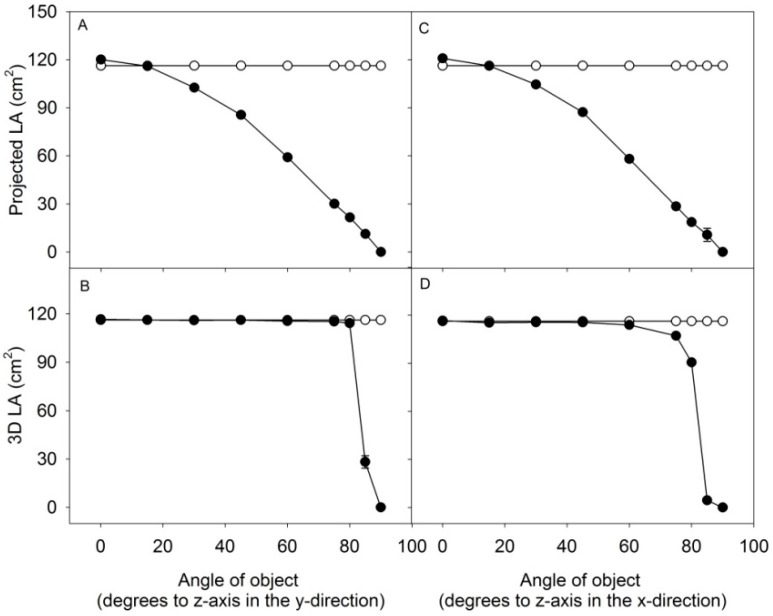
An object with defined area of 116.2 cm^2^ was tilted stepwise to the z-plane in the y- and x-direction and measured by the 3D scanner. Projected LA and 3D LA of the object was computed at different angles. Area of object (white dots), computed area of object (black dots). Values are average of three ± SE, with SE’s below 1 cm^2^ for most values.

A comparison between the estimate of 3D leaf area and projected leaf area (2D) was made by scanning a flat object with an area of 116.2 cm^2^ with the angle of the object towards the scanner increased incrementally in the y-direction (Figure 3A,B) or x-direction (Figure 3C,D). The surface of the object was estimated to 116.4 ± 2 cm^2^ at an angle of 0° for 3D area and to 118.9 ± 1 cm^2^ for 2D area. At an angle of 80° in the y-direction, the 3D area was 107.6 ± 3 cm^2^ and the projected area was 22.8 ± 1 cm^2^; a similar result was obtained when tilting the object in the x-direction. Above the angle of 80°, the 3D leaf area was incorrectly estimated, especially when the object was tilted in the x-direction. This was caused by insufficient scattering of light back into the camera. The small test demonstrate the advantages of 3D measurements, being sufficiently precise in predicting the area, even at increasing leaf-angles in both the x- and y-direction, compared to 2D which only allows the measurement of the projected leaf area.

### 2.2. Experiment 1: Testing a Potential Influence of the Projected Laser Line on Photosynthetic Activity

The 3D laser triangulation scanner is equipped with a NIR laser belonging to the laser class 1 M (940 nm), meaning that it is eye-safe to use in all conditions except when passed through magnifying optics.

In order to test whether a laser line of this power had any effects on the plant physiological performance, the PSII photochemistry of rapeseed plants was monitored continuously with a PAM fluorimeter (MONI-PAM, Walz, Effeltrich, Germany) for plants placed underneath the scanner. Half of the laser line was covered using black tape (TESA 4613, tesa A/S, Amsterdam, The Netherlands) and six plants were placed on the table so that three plants were exposed to the laser and three plants were not exposed to the laser. The system was set to measure every 30 min (00:00 h and 00:30 h) at a scanning speed of 20 mm·s^−1^. The measurements took 5 min from start to end, and the MONI-PAM was set to measure every 20 min (00:05, 00:25 and 00:45 h). The chlorophyll fluorescence measuring system consisted of six emitter-detector units (MONI-heads), each representing independent fluorimeters. Three MONI-heads were placed directly underneath the path of the laser line, and the three other MONI-heads were placed outside that path. Rapeseed leaves of similar age were fixed in the MONI-heads leaf clips, and measurements of photosynthetic active irradiation in the range of 400–700 nm (PAR, µmol·m^−2^·s^−1^) using the integrated quantum sensor, maximum photochemical efficiency of PSII during the dark period; F_v_/F_m_ = (F_m_ − F_0_)/F_m_, Quantum yield of PSII (Φ_PSII_); F´_q_/F´_m_ = (F´_m_ − F´)/F´_m_ and the electron transport rate (ETR) during the light period [14], were recorded continuously as described above. The intensity of the light saturating pulse was 1800 µmol·m^−2^·s^−1^ and the duration of the pulse was 0.8 s. The measurements were done on eight consecutive days. Every day at 10:00 h the MONI-heads were randomized, and a new part of the leaf was measured, and after four days the black tape was moved to cover the other 50% of the laser line in order to obtain a completely randomized design.

### 2.3. Experiment 2: Predicting Growth Parameters of Rapeseed by 3D Laser Triangulation

The experiment was conducted to develop a model from which it would be possible to predict destructive growth parameters of rapeseed by using the parameters obtained by 3D laser triangulation. The experiment on rapeseed was carried out from 24 September to 6 October 2013 (plant batch 1), and repeated from 8–29 November 2013 (plant batch 2) in a greenhouse located at the Department of Food Science, University of Aarhus (Aarslev, Denmark, Lat. 55°N). The light period was 20 h per day, supplemented by artificial light provided by high-pressure sodium lamps (SON-T agro, 600 W, Phillips, Eindhoven, The Netherlands) at 130 µmol·m^−2^·s^−1^ photosynthetic flux density. The temperature set point was 20 °C with opening of the vents at 25 °C. The plants were watered by flooding for a short period every second day.

Seeds of the rapeseed genotype “DH5” were sown in 11 cm pots containing peat and placed immediately on the table where the 3D scanner was mounted. The field of the 3D scanner was divided into ten longitudinal subfields (640 × 600 mm) with six pots in each subfield, distributed in two rows with three pots in each. The distance between the two rows was 80 mm and the distance between pots in the rows was 60 mm. Plants from each of ten subfields were harvested at growth stages defined by the number of leaves, to allow comparison of destructive and non-destructive measurements at different stages of plant development. The scanning measurements of each subfield were conducted with a scan velocity of the boom of 20 mm·s^−1^, giving a resolution of less than 1 mm in the scanning direction (y). Scans were carried out every hour over a period of three weeks.

The destructive harvests were carried out at 12:00 h, and all six plants from a subfield were harvested. For each plant, leaves were separated from the petioles, the number of leaves was counted (LN) and the total leaf area (LA) was determined using a leaf area meter (LI-3000, LI-COR, Lincoln, NE). Plant fresh weight (FW) was determined, plant material was dried at 70 °C for 24 h and plant dry weight (DW) was determined. The leaf area index (LAI) was calculated as total leaf area of the six plants per ground area of the subfield (1350 cm^2^). The average values per plant of the destructive growth measurements (FW, DW, LN, LA and LAI) were related to the estimated values of projected leaf area (cm^2^·plant^−1^), 3D leaf area (cm^2^·plant^−1^) and height (cm). The estimated values were calculated from the scanning measurements conducted in the middle of the dark period (00:00 h) the night before the destructive harvests. This time point was chosen based on earlier observations that variation in environmental conditions have least effect on the plant structure at this time of day, making the day-to-day measurements of growth most reliable. The calculated parameters were based on four out of the six measured plants to avoid two plants at the edge, which were expanding their leaves outside the plot. These two plants were manually removed from the 3D point cloud. The correlation analysis was carried out on plants with leaf numbers ranging from two to seven (including cotyledons) from six subfields out of ten from each batch (12 in total). In the last four subfields of both batches, the plants had too many overlapping leaves, or leaves were extending out of the plot.

### 2.4. Experiment 3: Validating the Model on Ten Rapeseed Genotypes

A screening experiment with ten rapeseed genotypes was carried out in order to validate the linear models on the rapeseed genotype “DH5” from experiment 2. The experiment was carried out from 26 July to 19 August 2013 with greenhouse climate set points similar to those for Experiment 2. The seeds of ten rapeseed genotypes, 0. Chuosenshu, 1. Cobra, 2. Expert OSR, 3. Palu, 4. Olympiade, 5. Major, 6. S13, 7. Resyn HO48, 8. Markus and 9. DH5, were sown in 11 cm pots containing peat and placed directly in ten subfields on the table where the scanner was mounted. The distance between the pots was similar to that in experiment 2. Scanning measurements of each subfield were conducted every hour at a scan velocity of 20 mm·s^−2^, as in experiment 2. At the final harvest 24 days after sowing (DAS), all plants from each subfield were harvested. For each plant, the leaves were counted (LN) and the total leaf area (LA) was determined using a leaf area meter (LI-3000, LI-COR, Lincoln, NE, USA). Plant fresh weight (FW) was determined, plant material was dried at 70 °C for 24 h and plant dry weight (DW) was determined. The linear models obtained in experiment 2 were used to calculate daily values for plant FW, DW and total leaf area, to generate growth curves for the 23 DAS until the 18 August, the day before harvest.

### 2.5. Data Analysis

Data analyses were carried out using the R-language stat package [15]. For experiment 2, the effects of independent variables of plant batch and measured scanning parameters on the dependent variables of the destructive harvest measurements were tested. There was no effect of plant batch number and therefore Pearson’s correlation coefficients were calculated for all plants and between all variables using the rcorr() function and corrected for multiple interferences using the Holm’s method. Linear correlations were calculated using the lm() function and the regression lines were fitted to the curves showing the relation between the scanning parameters and the destructive harvest measurements in Sigmaplot (ver. 11, Systat software, 2008).

In the screening experiment (Expt 3) the measurements of the destructive harvest after 24 DAS were analyzed using a one-way ANOVA with genotypes as main effect, and the average values were separated across the different genotypes using the glht() function to analyze for multiple comparisons using the Tukey correction (95% confidence intervals).

## 3. Results and Discussion

### 3.1. Photosystem II Efficiency of Rapeseed Was Not Affected by the Near-Infrared (NIR) Laser Line

**Figure 4:**
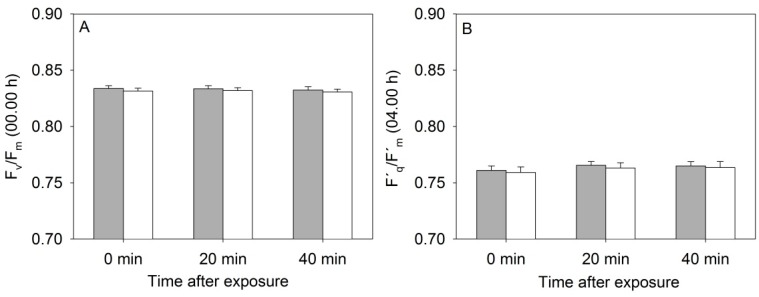
(**A**) Values of maximum photochemical efficiency of PSII (F_v_/F_m_) and (**B**) Quantum yield of PSII (F´_q_/F´_m_) in rapeseed plants exposed to the near-infrared laser line from the 3D triangulation scanner or placed in a control treatment. Control plants (grey bars) and scanned plants (white bars), *n* = 3 ± SE.

The diurnal measurements of PSII operating efficiency, (Φ_PSII_) and the electron transport rate (ETR) varied greatly in relation to the changing light conditions during the eight consecutive measurement days, and in relation to the actual light level at which the individual MONI-heads were positioned (results not shown). Furthermore, flashing with high light every 20 min during the night decreased the maximum photochemical yield of PSII (F_v_/F_m_), possibly because the leaves became less and less dark-adapted. However, F_v_/F_m_ was always above 0.8, which is in the range of F_v_/F_m_ values (0.79–0.84) shown not to affect plant yield [14]. As seen in Figure 4 there was no significant effect of the NIR laser line after 0, 20 and 40 min of exposure on the F_v_/F_m_ or Φ_PSII_ (F´_q_/F´_m_) values.

**Figure 5:**
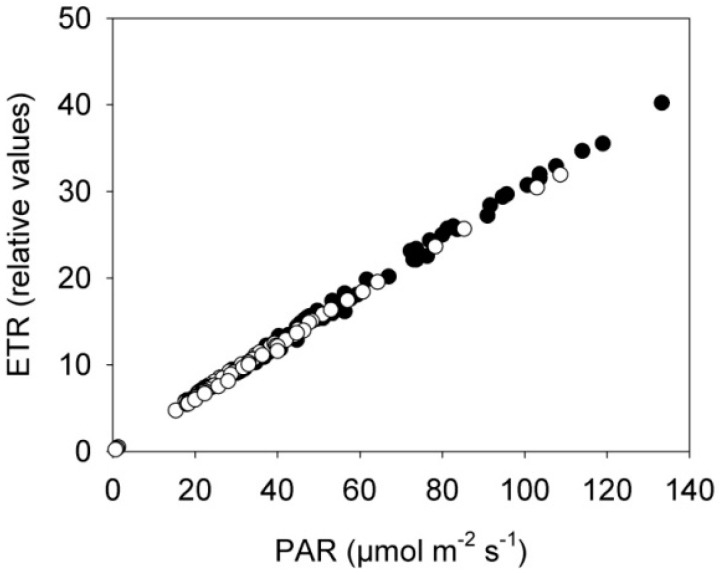
Correlation between the electron transport rate (ETR) and the photosynthetic active radiation (PAR) in rapeseed plants exposed to the near-infrared laser line from the 3D triangulation scanner or placed in a control treatment. Control plants (black dots) and scanned plants (white dots), *n* = 3.

These measurements were made continuously every hour throughout the eight days, but the figure only shows mean values from the two time points 00:00 h (beginning of dark period) and 04:00 h (beginning of light period) for the eight consecutive days in order to avoid effects of differences in light irradiance between consecutive days. The electron transport rate (ETR) was linear related to PAR under the low light conditions in the two treatments showing that photochemistry and photosynthesis of the plants was unaffected by the NIR laser line (Figure 5).

### 3.2. The Scanning Parameters Correlate with Growth of Rapeseed, but Total Leaf Area Is Underestimated 

Table 1 shows the correlation coefficients between the scanning parameters and the destructive growth measurements based on the dataset from 47 rapeseed plants of the cultivar “DH5”. The correlation coefficients were generally very high, ranging from r^2^ = 0.93 − 0.97 and with corresponding low *p*-value (<0.001) for all variables except when correlated with the height parameter and leaf number. The reason for this is that young rapeseed plants establish a rosette with older leaves at the base, increasing in size, and smaller, younger leaves developing in the center on a stem of increasing height up to a leaf stage of nine to 30 leaves, depending on the growth conditions. Plants that have no erect stem makes the height parameter from the scanner uninformative about the plant 3D structure. Furthermore, older leaves expanding continuously increase the variation in total leaf area of plants having the same number of leaves, height and weight.

**Table 1:** Correlation coefficients; r^2^ and adjusted *p*-values (Holm’s method) for correlation between the scanning parameters and destructive growth parameters in one genotype (DH5) of rapeseed. The destructive growth parameters are LAI (leaf area index), fresh weight (FW), dry weight (DW), leaf number (LN), leaf area (LA) and the scanning parameters are height, 3D leaf area (3D LA) and projected leaf area (Proj LA).

		LAI	FW	DW	LN	LA
Height	r^2^	0.86	0.93	0.93	0.89	0.94
	*p*	<0.01	<0.001	<0.001	<0.01	<0.001
3D LA	r^2^	0.93	0.97	0.97	0.88	0.97
	*p*	<0.001	<0.001	<0.001	<0.01	<0.001
Proj LA	r^2^	0.93	0.97	0.97	0.88	0.97
	*p*	<0.001	<0.001	<0.001	<0.01	<0.001

**Figure 6:**
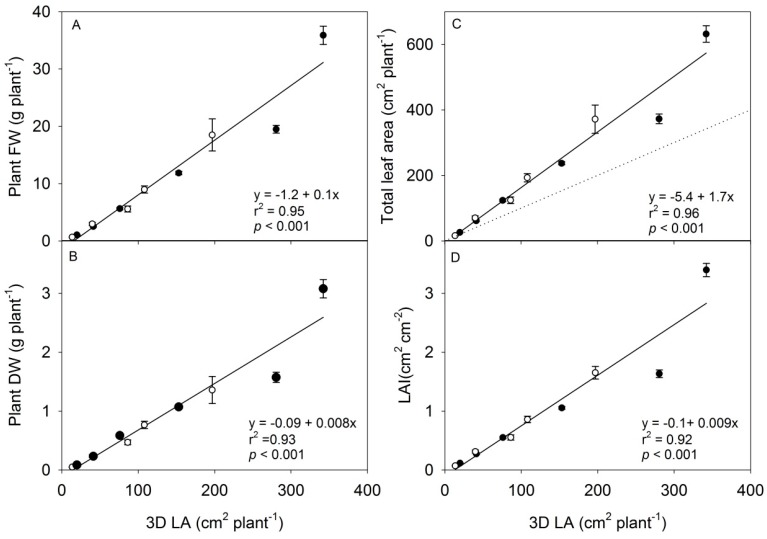
(**A**–**D**) Destructive measurements of plant FW, plant DW, total leaf area and Leaf area index (LAI) are shown in relation to the 3D leaf area parameter (3D LA) from the 3D triangulation scanner measurements, Experiment 1 (white dots), experiment 2 (black dots). N = 12, each data point display the mean of six plants ± SE. The stippled line shows the hypothetical 1:1 relationship between total leaf area and 3D leaf area.

The 3D leaf area had the highest correlation coefficient (Holm’s method) in predicting the biomass production of young rapeseed plants in terms of DW or FW (r^2^ = 0.97, *p* = 0.001) compared to the projected leaf area (Table 1). Therefore, it was decided to base the prediction of plant weight and leaf area on the parameter of 3D leaf area (Figure 6). As seen in this figure, the fitted linear correlation fits well to the data up to a 3D leaf area of 200 cm^2^·plant^−1^ whereas the last two points are not well fitted, showing that the method is not reliable for larger size plants. It is further seen that the scanner underestimated the total leaf area; the 3D leaf area was 50%–90% of the measured leaf area depending on the size of the plant (Figure 5C, stippled line). The main reason for this underestimation is the overlapping of leaves, which increases in time and with developmental stage, but also because any leaf surfaces with angles larger than 85° to the z-axis would not be detected (see Figure 2). Overlapping leaves and leaves expanding out of the plot are common limitations of non-invasive methods used within the phenotyping community [4]. In the present set-up, the maximum scan width, the distance from the scanner and the distance between the pots limited the number of plants that could be measured in each subfield. Despite of this, we found that there were good correlations using the 3D point cloud monitored from one angle on the rapeseed plants with leaf areas ranging from 2.5–400 cm^2^ and shoot dry weight ranging from 0.01 g–1.5 g per plant. This was in the same range as seen for individual rapeseed plants monitored by light curtain arrays (LCs) from one to 16 different angles [4], and in cereals monitored individually from three different angles using a conveyor based imaging station [2]. It has been shown that 3D laser scanning with the use of at least three different angles can estimate above-ground biomass of juvenile trees in the range from 3 to 12 g [16], and a set-up with several 3D triangulation scanners placed at different angles could possibly improve the estimation of larger plants. A similar approach was shown to improve the accuracy of estimation by light curtain arrays [4].

The leaf area index (LAI) is an important parameter for defining canopy structure, light use efficiency and in predicting primary production. It follows a near-linear relationship with crop growth rate and net photosynthesis up to a species-specific optimum LAI value, which often approximates three [17]. Our results show that LAI can be well estimated up to a value of two. At this value, crop growth rate is directly related to light interception over the whole leaf area and is not limited by overlapping leaves.

### 3.3. 3D Laser Triangulation Identified Phenotypic Variation in Rapeseed

**Figure 7:**
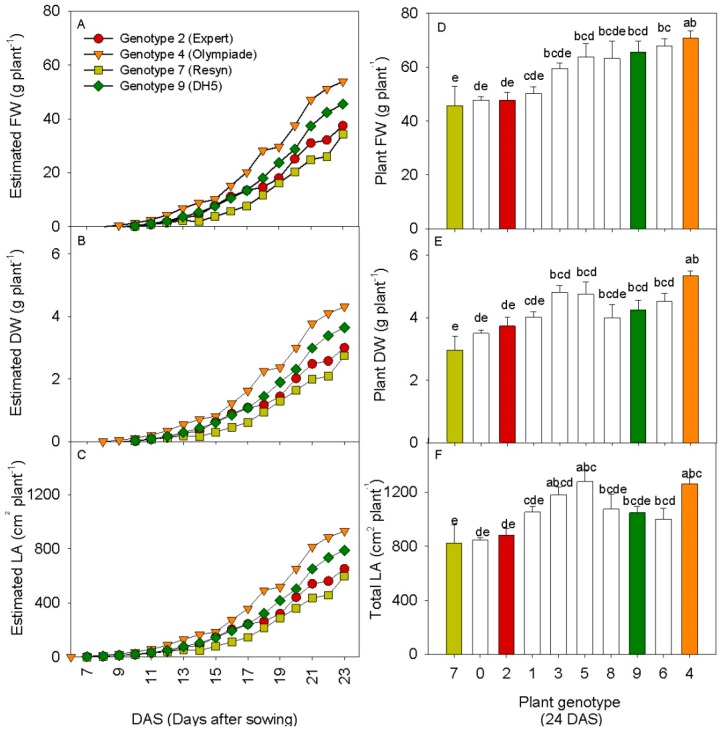
(**A**–**C**) Growth curves from 6–23 DAS for estimated values of plant FW, plant DW and total leaf area in four selected genotypes of rapeseed; (**D**–**F**) Destructive harvest values of plant FW, plant DW and total leaf area in ten genotypes of rapeseed at 24 DAS. For the destructive parameters the values are mean of six plants ± SE, letters represent significant differences between means (*p* < 0.05). The colored bars represent the same genotypes.

Scanning measurements taken every hour for 23 days after sowing (DAS) on ten different rapeseed genotypes with similar architecture were transformed to plant FW, plant DW and total leaf area based on the linear correlation between the destructive plant growth measurements and the estimated 3D leaf area from the scanner in experiment 2 on one of the cultivars (“DH5”). Growth curves for plant FW, plant DW and total leaf area of four selected genotypes of rapeseed are shown in Figure 7A–C.

For the destructive harvest values, the statistical analysis revealed significant differences between the cultivars for all three growth parameters, and the result of the multiple comparisons of means tests is shown as letters above the bars (Figure 7D–F). The results show that despite large variances in between the cultivars, only some of them were significantly different. It is clear from the predicted values of weight and leaf area after 23 DAS that the 3D laser scanning successfully distinguished the two cultivars 7. Resyn and 4. Olympiade, which were also significantly different in terms of destructive growth parameters after 24 DAS. Furthermore, the 3D scanning also placed the two genotypes 2. Expert and 9. DH5 in between the significantly different genotypes, and in the same sequence, using the mean values from the destructive harvest. The predicted values and the harvested values from the four genotypes formed linear correlations with r^2^-values ranging from 0.94–0.99 (results not shown). However, when including all ten genotypes, the r^2^-values dropped due to the insignificance of the destructive harvest results. This underlines, not surprisingly, the point that non-invasive methods like 3D laser triangulation do not predict genotypic differences in plant growth *better* than destructive methods. However, the temporal resolution is improved, as was also shown for another image-based approach [18]. Further, it demonstrates that 3D laser triangulation from just one perspective is a valid alternative to destructive harvest measurements.

### 3.4. Diurnal Patterns in Scanning Parameters Contain Information on Changes in 3D Plant Structure 

**Figure 8:**
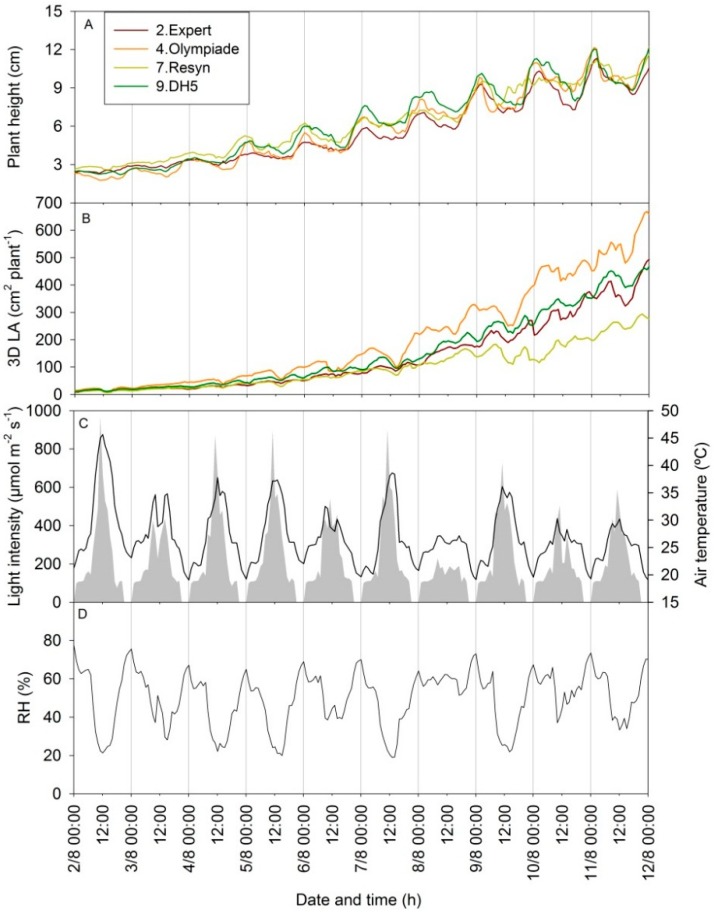
(**A**,**B**) Growth curves from 7–16 DAS (02.08.2013–12.08.2013) for the 3D scanner measurements of height (mm) and 3D LA (cm^2^·plant^−1^) in four selected genotypes of rapeseed; (**C**) Light intensity (grey area) and air temperature (black line); (**D**) Relative air humidity (% RH) in the same period, shown as mean value per hour.

The scanning parameters of height and 3D leaf area oscillate in a diurnal pattern (Figure 8). The magnitude of these oscillations depends on the climate conditions and is caused by a combination of nyctinastic leaf movements and diurnal variation in leaf and stem expansion, and these may differ a lot between plant species [19]. In Figure 8, the values of plant height and 3D leaf area (cm^2^·plant^−1^) are shown for the four genotypes of rapeseed on an hourly basis from 7–16 DAS, 2–12 August (Figure 8A,B), with the corresponding climate parameters (Figure 8C,D). The amplitude of the diurnal measurements varied between the different days, but these differences were not directly an indicator of differences in the actual increase in plant size during the 24 h day from 00:00 h to 00:00 h the following night. It was seen that a clear warm day (e.g., 7 August) with high light intensity and low air RH resulted in a large drop in the 3D leaf area by midday in all four genotypes and that the genotype with the highest growth rate experienced the largest drop (4. Olympiade). However, the corresponding diurnal pattern in plant height was not directly related to this drop in 3D leaf area, as similar oscillations in the plant height measurements were seen on 8 August, where the diurnal oscillation in 3D leaf area was less pronounced. The temporal variation in the non-invasive measurements may illustrate that circadian movements of leaves from a horizontal to a more vertical position at night [20] alter the 3D structure of the plant, causing differences in the plant height measurements throughout the day, and that low light intensities reduce the circadian leaf movements [21]. These circadian leaf movements may also have increased the area of leaf surface with an angle larger than 85° to the z-axis, beyond the detection limit of the scanner.

We suggest that the drop in 3D leaf area on the 7 August and the other days with high light intensities was an effect of light avoidance, induced by the low humidity and a corresponding high vapor pressure deficit (results not shown), closing leaf stomata, decreasing leaf turgor, and thus increasing the leaf angle, so that a larger surface area was beyond the detector’s detection limit. It is tempting to suggest that these genotypic-specific leaf movements, related to plant water status, explain some of the variation in growth, but it is beyond the present paper to validate this.

## 4. Conclusions

The development of methods to phenotype plants with a high frequency and revealing short and long term growth patterns is of importance for research. We have demonstrated that 3D laser triangulation is a valid tool to estimate growth and structural information of plants, and can be used for active “sensor-to-plant” approaches. This enables frequent measurement and the possibility to study fast responses and adaptations of plant structure to fluctuating environmental conditions. The laser line which is projected on plants, in order to compute height profiles, does not affect the plant photosynthetic performance at the frequency of measurements used. The methodology can give a rapid estimate of plant weight and leaf area in contrasting genotypes of rapeseed with similar architecture based on linear correlations from one genotype. The estimation of plant growth parameters was sufficiently accurate for rapeseed plants up to the 7th leaf stage, but these results may be improved by increased spacing of the plants to avoid leaf-overlap, and by increasing the height, and hence scan width, of the scanner to allow for even taller plants to be measured. Scanning from more perspectives could improve the accuracy, but with a need for more algorithms. In addition, the analysis of measurements in high temporal resolution could reveal information on crop responses to abiotic stresses, showing how plants alter their 3D canopy structure to optimize light interception and avoid water loss under natural light, drought and temperature stresses.
